# Contribution of Rainfall on Rooftop Rainwater Harvesting and Saving on the Slopes of Mt. Elgon, East Africa

**DOI:** 10.1155/2020/7196342

**Published:** 2020-07-18

**Authors:** Barasa Bernard, Asaba Joyfred

**Affiliations:** Department of Geography and Social Studies, Kyambogo University, P.O. Box 1, Kyambogo, Kampala, Uganda

## Abstract

Despite the achievements reported from using rainwater harvesting systems, the contribution and drawbacks that affect their usage in mountainous landscapes have received little attention. The uptake and usage of domestic rooftop rainwater harvesting systems (RRWHS) in developing countries is on the increase due to increasing water scarcities. We explored the effect of rainfall variability on water supply and the downsides of using the systems by rural households in Uganda. The objectives were to assess the variability of rainfall (1985–2018), categorise RRWHS used, and examine the influence of slope ranges on the placement of systems and also to quantify the harvested and saved rainwater and establish the factors that affected system usage. Rainfall variability was assessed using a Mann–Kendall test, while system contributions and drawbacks were examined using socioeconomic data. A representative of 444 households were selected using a multicluster sampling procedure and interviewed using semistructured questionnaires. Findings revealed that the months of March, April, September, August, and October experienced an upward trend of rainfall with a monthly coefficient of variation between 41 and 126%. With this, households responded by employing fixed (reinforced concrete tanks, corrugated iron tanks, and plastic tanks) and mobile RRWHS (saucepans, metallic drums/plastic drums, jerrycans, and clay pots). At the high altitude, households deployed mostly plastic jerrycans and industrial plastic/metallic drums to harvest and save water. Overall, the mean annual volume of rainwater harvested on the slopes of Mt. Elgon was 163,063 m^3^/yr, while the potential to save water ranged from 4% to 7% of the annual household water demand. The factors that hindered the deployment of RRWHS to harvest and save water were high operational costs, price fluctuations, unreliable rainfall pattern, inadequate funds, and limited accessibility. The rainfall received if well-harvested and saved can redeem households of water insecurity, though there is an urgent need of subsidies from the government to increase accessibility of the systems.

## 1. Introduction

Water is vital for the survival of mankind on Earth. The demands for water may also vary from plants and animals among others [1]. This water can be accessed through several means such as well, boreholes, taps, rivers, lakes, and rain for use of both domestic and commercial purposes. Domestically, water can be used for cooking, cleaning, drinking, and irrigation among others, while, commercially, the water can be used as raw materials in industries, large scale irrigation, municipal water supplies among others [2]. Although water sources exist and seem to be accessible to households in many parts of the world, rainwater is the most effective alternative and the cheapest to access [3].

Rainwater harvesting systems are fixed on permanent houses although some are mobile that are only brought out of houses during the rain to harvest water. In most cases, for both, domestic rooftop rainwater from the catchment area is guided by gutters to tanks or storage medium where the water is saved and abstracted when needed using taps or other tools such as cups or other containers among others. This water is used by the households for many purposes like cooking and washing [4]. However, the usage and saving of harvested rainwater depend on the climate where a household is located. For example, many households in the tropics have installed rainwater harvesting systems on their houses to meet water scarcity challenges especially during the dry periods in the year. During the scarcities, households that are not connected with consistent water supplies lose a lot of time and energy trying to access the water sources [5]. Challenges that could limit most households to store rainwater for a longer period can include household size, type of tool used, the storage capacity of the tank, size of the catchment area, and rainfall pattern [6]. As a result, households that would want to store water up to the dry season have opted for the installation of concrete tanks, plastic tanks, and corrugated tanks because of high storage capacities and free from high evaporation effects [7].

Despite the importance of installing rooftop rainwater harvesting systems on houses to harvest water, few households have adopted the strategy in many areas faced with high rainfall variabilities. For example, in many developing countries, especially in Africa, the drawbacks that could limit households from acquiring and using the tools include high poverty rates, limited accessibility to the systems, type of dwelling, accessibility challenges especially for those staying in hilly areas, and lack of information on the suitability of rainwater harvesting systems [8]. However, the broader context of these challenges is as a result of limited government support in promoting rainwater harvesting tools; for instance, some countries have not developed manuals to guide masonry to construct the systems. There are no subsidies and policies or strategies that could support or guide the subsector to thrive [9].

In addition, rainfall variability is also a challenge to the functionality of rainwater harvesting systems. The characteristics of horizontal variability of rain can be presented as a fraction rain cover or probability distribution. Rainfall variability has a significant contribution to the amount of water that can be saved by rooftop rainwater harvesting systems. The rains received like in the East African region are highly variable over space and time. This variability (either interannual or intra-annual) is often caused by the El Nino Southern Oscillation (ENSO) that influences the Intertropical Convergence Zone (ITCZ) which induces ocean dynamics that affects the seasonality of rainfall received [10]. As a consequence, this has impacted the amount of water stored, and so limited its use; thus the sustainability of the installed rainwater harvesting systems in the region. Despite these limitations, the use of these systems has proven to be vital to support the livelihood of rural communities especially for those that have embraced the technologies partly because they have saved time and energy required to access water from the alternative sources [11].

This study recognises that several studies have been conducted in the arena of rainwater harvesting, for example, the potential of rooftop rainwater harvesting [12, 13], the impact of rainwater harvesting on climate change adaptation [14], increment in water supply [15], assessing the potential of rainwater harvesting systems in institutions [16], rainwater harvesting in modern cities [17], and their performance [18]. Nonetheless, little attention has been paid to the contribution of deploying domestic rooftop rainwater harvesting systems to store and save water in mountainous landscapes dominated by rural households. Also, rarely, meteorological data, socioeconomic household survey, and census population datasets have been fused to understand the contributions of rainwater harvesting abilities to store and save water. Therefore, the purpose of this study was to explore the effect of rainfall variability on water supply and the downsides of using the systems by rural households in Uganda. The objectives were to assess the variability of rainfall (1985–2018), categorise RRWHS used, and examine the influence of slope ranges on the placement of systems. Also, quantify the harvested and saved rainwater and establish the factors that affected system usage on the slopes of Mt. Elgon located in Eastern Uganda.

## 2. Materials and Methods

### 2.1. Description of the Study Area

**Figure 1:**
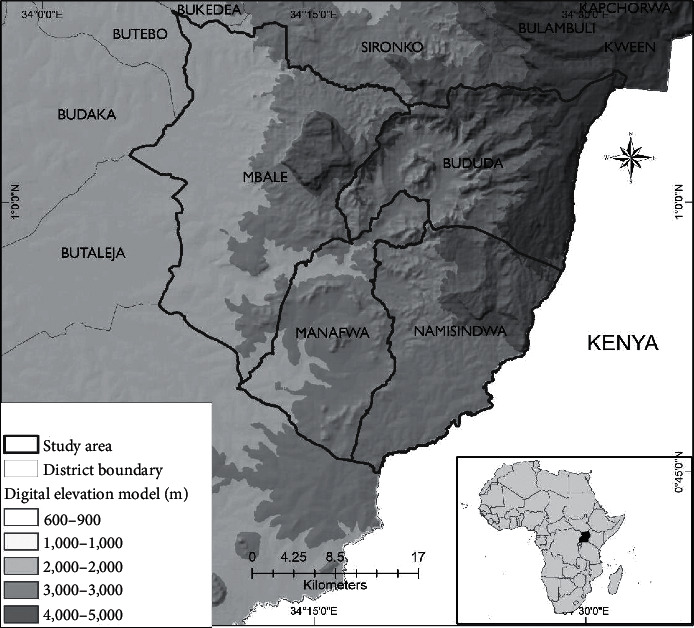
Slopes of Mt. Elgon in Uganda.

This study was conducted on the slopes of Mt. Elgon in Uganda. Although the mountain is transboundary, the Ugandan side was considered due to logistical limitations. The extinct volcano is shared between Uganda and Kenya (Figure 1). It is the oldest and largest volcanic highland in East Africa. It stands at 4,320 meters and 80 km in diameter. The upper reaches of the mountain are degazetted as national parks while the rest is inhabited. The studied districts that lie on the slopes of the mountain were Mbale, Namisidwa, Manafwa, and Bududa. These were selected because of reported shifts in seasonal profiles due to changes in climate [19]. Therefore, this study sought to find out the contribution of rainfall on domestic rooftop rainwater harvesting with increasing times of water scarcities. The districts sampled experience a tropical equatorial humid climate which is described by a bimodal rainfall pattern. They receive a mean annual rainfall of about 1,400 mm and 1,800 mm. The long rainy period is between March and May, while the short rainy months are October and November, while the mean annual temperature varies from 14°C to 25°C. The topography of the mountain is rugged and characterized by steep and gentle slopes, whereas the soils are predominantly of volcanic origin (composed of mainly clay, loam, and sandy soil types). However, in terms of survival, the most dominant source of household income is sale of crops and livestock. The major crops cultivated include coffee, banana, beans, and maize, while the major livestock kept are cows, goats, pigs, sheep, etc.

### 2.2. Collection of Household and RRWHS Datasets

This research was conducted between July and August 2018. In the selected districts, two subcounties were purposively chosen based on the advice of the district officials in terms of accessibility, water scarcities, and uptake of domestic rooftop rainwater harvesting systems. At the subcounty level, four parishes were selected and, in each, three villages were also purposively selected following the same criterion. Heads of families (female or male) were the targeted persons for this study. The households were selected using a multicluster sampling procedure. This technique helped to form a more efficient probability sample in terms of monetary and time resource [20]. Based on UBOS [21], census results, the population of Mbale was 568,800, Namisidwa had 226,100, Manafwa with 171,300, and Bududa had 259,800 people. The sample size was determined using Israel [22] sample determination procedures. Therefore, a total of 444 households were sampled. This sample size was sufficient to detect differences and associations but also have a comprehensive understanding of elements under investigation [23]. From each district, 111 respondents were selected with the guidance of local village leaders using membership lists to cluster households for investigations. The selected respondents were each subjected to face-to-face oral interviews using semistructured questionnaires from their homesteads.

The key informants were also purposively selected for interviews on the advice of the District Chief Administration Officer who oversees the implementation of government programs. The sample size included agricultural officers, water officer, wetlands officer, community development officer, and planning officer. These were interviewed also on a one-on-one basis from their offices. The procedure for their selection was based on their job responsibilities, knowledge and experience on household water access and use, and domestic rooftop rainwater harvesting. Similarly, focus group discussions were however conducted at the parish level. In each parish, three members were invited to convene at a commonly known meeting point. The sub-county headquarter premises were the most preferred because of accessibility and mobilisation reasons. One focus group discussion was held in each district to seek their opinions on rooftop rainwater harvesting. These participants were purposively selected by their parish leaders for those who had or not installed/used the rural rooftop rainwater harvesting systems. Each group was composed of between 12 and 14 members including youths, females, males, leaders, and elders. This number was considered manageable due to study resources limitations. However, understanding the quality and suitability of water harvested was beyond the scope of this study. The collected data were captured and analysed using Statgraphics software 18 because it is capable of handling data analysis for visualization and qualitative and quantitative analysis.

### 2.3. Influence of Altitude on the Placement of Rural Rooftop Rainwater Harvesting Systems

The locations of the systems were mapped using calibrated handheld Garmin eTrex Global Positioning Systems at an accuracy of 3 m. The coordinates were recorded in notebooks and also saved on the device for downloading as a risk control measure. The collected point features were added and geo-processed in a spatial environment. ArcGIS software version 10.4 was used to classify the altitudinal gradient (meters above sea level) ranges into four classes, using equal interval classification mode. The upper limit value ranges were used to define class boundaries of altitude as high (1744 m), moderate (1421 m), low (1392 m), and very low (1197 m). In addition, for each interviewed household, the catchment roof area was determined using a measuring tape and level and ladder.

### 2.4. Rainfall Variability Analysis

**Figure 2:**
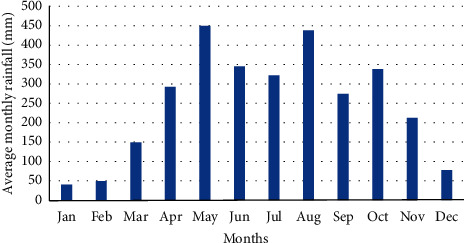
Mean monthly annual rainfall between 1985 and 2018.

Rainfall data is a key ingredient in the assessment of domestic rural rooftop rainwater supply. This data was attained from the Uganda National Meteorological Authority (UNMA) from 1985 to 2018, from five weather stations. However, few missing data gaps existed especially for the years 1986 (2 months) and 1987 (1 month) equivalent to 4% out of the data collected. These were filled using the most frequent method [24].  Figure 2 shows the average monthly annual rainfall between 1985 and 2018. The months that experienced the highest amount of rainfall were May and August. This was followed by June and October, while the lowest amount of rainfall was experienced in January, February, and December.

The variability of rainfall was assessed using a Mann–Kendall test [25, 26]. This test is widely used to analyse trends of hydrometeorological variables [27]. It was evaluated using a *Z* test and the statistical level of significance (0.05). The Mann–Kendall test was computed as follows:

(1) $$\begin{array}{c} S=\sum_{k=1}^{n-1}\sum_{j=k+1}^{n}sgn\;\left(xj-xk\right). \end{array}$$

The test is used to a time series *kX*, which is ranked from *k* = 1, 2, 3,…, *n*−1, which is ranked from *j* = *i* + 1, *i* + 2, *i* + 3…*n*. Each of the data points *x j* is taken as a reference point as follows:

(2) $$\begin{array}{c} Sgn\left(xj-xk\right)=+1\;\mathrm{if}\;xj-xk>0,\\ =0\;\mathrm{if}\;xj-xk=0,\\ =-1\;\mathrm{if}\;xj-xk<0. \end{array}$$

The variance of S is determined by

(3) $$\begin{array}{c} \text{VAR}\left(S\right)=\;\frac{N\left(N-1\right)\left(2N+5\right)-\sum_{k=1}^{n}t_{k}\left(t_{k}-1\right)\left(2t_{k}+5\right)}{18}, \end{array}$$

where *n* is the number of tied (zero difference between compared values) groups and *t*_*k*_ is the number of data points in the *k*^*th*^ tied group. For *n* greater than 10, the standard normal variate *z* is calculated by using

(4) $$\begin{array}{c} Z=\left\{o\begin{array}{c} \frac{S-1}{\sqrt{\text{VAR}\left(S\right)}\;\;\;}\;\;\mathrm{if}\;S>0\\ \frac{S+1}{\sqrt{\text{VAR}\left(S\right)}}\;\;\mathrm{if}\;S=0\\ \text{if}\;S<0 \end{array}\;\right\}. \end{array}$$

The positive *z* value indicates an increasing trend while a negative *z* value indicates a decreasing trend. When testing two-sided trends at a selected level of significance, the null hypothesis of no trend is rejected if the absolute value of *z* is greater than *z*_a/2._

Sen's slope estimator was also evaluated to estimate the extent of trend:

(5) $$\begin{array}{c} T_{i}=\frac{x_{j}-x_{k}}{j-k}\text{for}\;i=1,2,3,\ldots ,N, \end{array}$$

where *X* and *X*_*k*_ are the data values for *j* and *k* times of a period, where *j* *>* *k*. The slope is estimated for each observation. Median is computed from *N* observation of the slope to estimate Sen's slope estimator as follows:

(6) $$\begin{array}{c} Q_{i}=T_{N+1/2\;}\text{for}\;N\text{ is odd}\\ =\frac{1}{2}\left[T_{N+/2\;}T_{N+1/2\;}\right]\text{ for}\;N\text{ is even.} \end{array}$$

When the *N* slope observations are shown as Odd, Sen's Estimator is computed as *Q*_med_ = (N + 1)*/*2 and for even times of observations, the slope is estimated as *Qmed* = [(N/2) + ((N + 2)/2)]/2. The two-sided test is carried out at 100 (1–*α*) % of the confidence interval to obtain the true slope.

Rainfall variability was tested using the coefficient of variation (CV). The CV was computed as follows:

(7) $$\begin{array}{c} \text{CV}=\frac{\sigma }{\mu }\times 100, \end{array}$$

where CV is the coefficient of variation, *σ* is the standard deviation, and *μ* is the mean precipitation. CV is used to classify the degree of variability of rainfall events as less (CV < 20%), moderate (20 < CV < 30%), high (CV > 30–40%), and very high (CV > 40–70%) which shows extremely high interannual variability of rainfall.

### 2.5. Household Capacity of Rainwater Harvested

Household annual water demand (PWD) and rooftop catchment area (A) data were captured during the socioeconomic field survey. The quantity of rainwater harvested was aggregated at the district level (Manafwa, Namisindwa, Mbale, and Bududa) (VR). The household population of each district from the national census database was considered in this computation. The sampled households roofed their houses using corrugated iron sheets with a smooth surface. The rooftop runoff coefficient (C) was 0.9 [28]. It was assumed that this runoff coefficient catered for a loss of 10% of the rainwater that was discarded for roof cleaning and possible evaporation. Therefore, the volume of water harvested in each district was computed using the following equation:

(8) $$\begin{array}{c} \text{VR}=\left(R\;x\;A\;x\;\frac{c}{1000}\right). \end{array}$$

The district annual water saving (APPWS) was compared to the volume of water that could be harvested. This was computed using the following equation:

(9) $$\begin{array}{c} \text{APPWS}=100\frac{\text{VR}}{\text{PWD}}, \end{array}$$

where APPWS is annual potable water saving for each district (%), VR is the annual volume of rainwater harvested in each district (m^3^/yr), and PWD is annual potable water demand (m^3^/yr).

## 3. Results

### 3.1. Variability of Rainfall and Installation of Rooftop Rainwater Harvesting Systems

Findings in Table 1 show that the highest annual rainfall received between 1985 and 2018 was experienced in May and August. The lowest observations were experienced in January, February, and December. This result further reveals that a monotonic rainfall upward trend was experienced in March, April, September, August, and October although the downward trend was seen in February. This finding is significant at *P* ≤ 0.05. The average monthly CV alternated between 41 and 126%, whereas Sen's slope trend increased in October, August, September, and April (Table 2). Conversely, the negative trend of the slope was observed in February, while a relatively slight upward trend was seen in March, July, and November that ranged from 3.03 to 3.31. Rainfall data was characterized by more of the upward trend than the downward trend.

**Figure 3:**
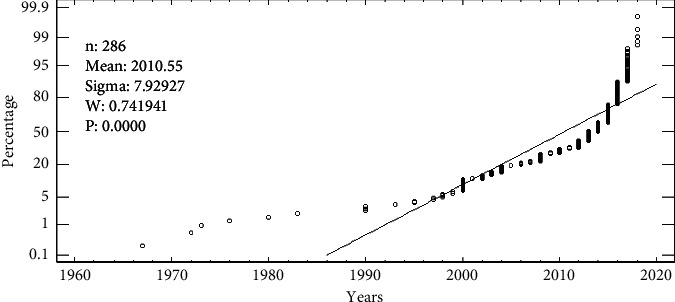
Installation of rooftop rainwater harvesting systems from 1960 to 2019.

As per the installation of systems concerning the variability of rainfall identified, the household's deployment of rooftop rainwater tools is on the increase. The uptake is due to the account that the rains had become unreliable.  Figure 3 shows the installation of rooftop rainwater harvesting systems from 1960 to 2019. This study shows that the rainwater harvesting system's uptake increased from 2000 (20%) to 2018 (80%) than before as reported by the households. Similarly, the key informant findings showed that the households installed the rainwater harvesting tool to cope with increasing scarcities of water and these were mostly installed in the dry season (December–February) to fill up with water in the rainy period (March–May, June–August, and September–November). The most installed rainwater harvesting systems were reinforced concrete tanks, corrugated iron tanks, and plastic tanks due to reliability and durability. However, the participants in the focus group discussions reported that the harvested water was not good for drinking unlike washing and cleaning because it had silt from catchment areas.

### 3.2. Categorisation of Domestic Rural Rooftop Rainwater Harvesting Systems

The installed domestic rural rooftop rainwater harvesting systems were categorised as fixed and mobile. Fixed systems were permanently placed beside the houses, while the mobile ones were mostly put into use whenever it rained. The fixed systems included reinforced concrete tanks, corrugated iron tanks, and plastic tanks; however, the mobile systems comprised saucepans, metallic drums/plastic drums, jerrycans, and clay pots.  Table 3 illustrates the typology and description of installed rural rooftop rainwater harvesting systems. Many of the households (74%) during the focus group discussions reported having roofed their houses using corrugated iron sheets. The adoption and use of fixed systems were attributed to durability and safety but also had high water storage capacities and weather resistant.

The altitudinal gradient of Mt. Elgon can be described as convex and concave as observed. Using the altitudinal values, the sampled habitable gradient range was categorised as high (1744 m), moderate (1421 m), low (1392 m), and very low (1197 m). At the high-altitude range, the gradient influenced the installation and use of plastic jerrycans and industrial plastic/metallic drums, while, at the moderate the level, the plastic tanks were the most preferred (Table 4). From the lower gradient range, the most used domestic rural rooftop rainwater harvesting systems were plastic jerrycans, industrial metallic/plastic drums, plastic tanks, and saucepans. Generally, the fixed rainwater harvesting systems were the most preferred at the high altitudinal gradient range, whereas the mobile systems were opted at the moderate and lower altitudinal gradient ranges.

### 3.3. Amount of Rainwater Harvested by Households on the Slopes of Mt. Elgon

In this study, all the interviewed households roofed their houses using corrugated iron sheets. The mean minimum roof catchment area was 206 sqm, while the mean maximum was 8,796 sqm (Table 5). From the observations, the measured roofs were hip-roofed. However, the participants in the focus group discussions reported that the type of roof and roofing design were not significant factors in the adoption of any domestic rooftop rainwater harvesting system installed or utilised. The description of rainwater harvesting tanks was based on fixed systems. These systems were corrugated iron sheet tanks, reinforced concrete tanks, and plastic tanks. For the recorded tanks, the average tank storage capacity was 307 litres. The key informants revealed that these systems increased water supplies due to big tank storage capacities. The household water demand for the harvested rainwater was mainly for potable and nonpotable purposes. The computed mean demand for rainwater was 84 litres per day throughout the year. On the seasonal basis, the installed tanks were not able to meet the household water demands for 74 days in the dry season, while 25 days in the wet season. What is worthy to note here is that, in the dry season, the used rainwater tanks were only able to provide water for 15 days, while in the wet season the water existed for 63 days.

The computed average district rainwater demand was 84 L/d per capita. This ranged from 71.3 L/d per capita to 94 L/d per capita (Table 6). In the studied case, the mean annual volume of rainwater harvested was 163,063 m^3^/yr. The annual volume of rainwater harvested ranged from 136,379 m^3^/yr to 195,121 m^3^/yr. Interestingly, the most striking result to emerge here is that the districts of Mbale and Namisidwa had relatively higher amounts of annual rainwater harvested compared to Bududa and Manafwa. Mbale and Namisidwa districts are located in the moderate and low altitudinal gradients. The potential to save water in the four districts ranged from 4% to 7% of the annual water demand with an average of 1469 L/y per capita. This result indicates that a person can save 4 litres of water daily. The participants in focus group discussions explained that “most of the rooftops had turned from white colour to dark brown (rusty) and trapped a lot of dust during the dry season that constrained their effort to save and use water.”

### 3.4. Factors Hindering the Use of Domestic Rural Rooftop Rainwater Harvesting Systems

**Figure 4:**
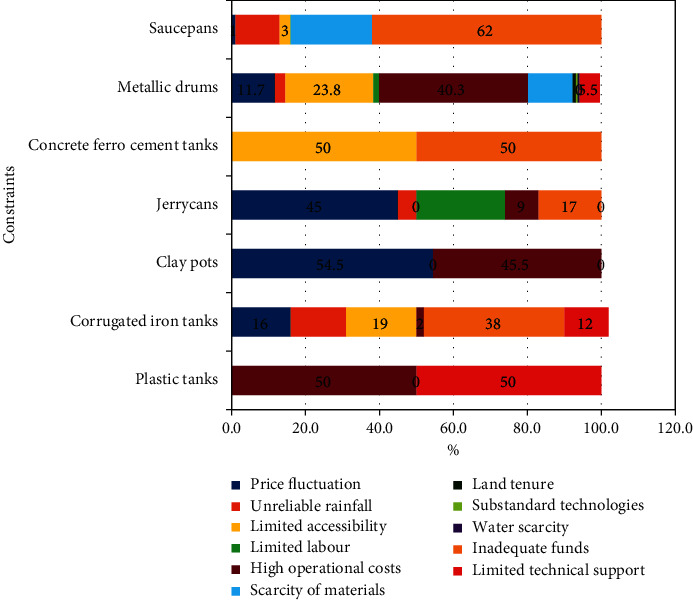
Factors that hindered the use of domestic roof rainwater harvesting systems (*N* = 444).

The factors affecting the use of domestic rural rooftop rainwater systems are holistic. These ranged from acquisition, installation, use, and maintenance.  Figure 4 presents the factors hindering the use of rural-rooftop rainwater harvesting systems. Based on the categorisation of the studied RRWHS, the factors that affected the fixed rainwater harvesting systems were high operational costs, limited technical support, limited accessibility, inadequate funds, price fluctuations (price increase), and unreliable rainfall. On the other hand, the mobile facilities were affected by the scarcity of system component materials, limited labour, price increases, and unreliable rainfall. Likewise, during the focus group discussions, most of the participants articulated that “poverty constrained most of the farmers to buy bigger water tanks to store water and use it during the dry seasons.” Besides, “most farmers used 20 litres of jerrycans that could not store water for long.”

## 4. Discussion

### 4.1. Variability of Rainfall on the Slopes of Mt. Elgon

The results of this study showed that the amount of rainfall received was variable. This can be explained by the high values of the coefficient of kurtosis and skewness in February. High rainfall fluctuations were experienced between 2010 and 2014. The lowest mean rainfall values were observed in January, February, and December. All the months recorded a CV of more than 30% indicating a high variability of rainfall over the slopes of Mt. Elgon. These months are characterized by water shortages. This could be caused by anomalously high pressure in the west of the Indian which is responsible for drought events in Elgon [29]. El Nino Southern Oscillation (ENSO) influences the Intertropical Convergence Zone (ITCZ) that induces ocean dynamics that affects the seasonality of rainfall [10]. Bomuhangi et al. [19] add on that the changes in climate variability are responsible for high intensity and frequency of rainfall in Elgon region. In short, the East African region is prone to unreliable rainfall due to changes in climate [30]. The months of May and August experienced the highest amount of rainfall received in the studied period. Relief of Mt. Elgon influences the amounts of rainfall received.

An upward trend of rainfall was recorded in March, April, September, August, and October. The trend of rainfall was significant in both long (March and April) and shorter wet season (October and September). What is important is that this trend shows peak months of both wet seasons which could trigger an intensification of rural rooftop rainwater harvesting but also indicate the potential for water-based activities (portable and nonportable). However, in low lying areas of East Africa, April is the only month that posts a significant rainfall trend [31]. Therefore, the amounts of rainfall received on the slopes of Mt. Elgon can be described as erratic and torrential with high rainfall depth that warrants the use of rooftop rainwater harvesting systems for effective water supply alternative.

### 4.2. Categorisation of Rural Rooftop Rainwater Harvesting

The rainwater harvesting systems used by the households can be classified as fixed (all year usage) and mobile (day/seasonal usage). All year used technologies included reinforced concrete tanks, corrugated iron tanks, and plastic tanks, while day or seasonal were saucepans, metallic drums/plastic drums, jerrycans, and clay pots. Movable facilities had temporal conveyance materials and small storage capacities, while fixed systems were permanently placed beside the buildings for easy accessibility and monitoring. Rainfall deficits explain the use of multisystems to harvest rooftop rainwater but also domestic water conservation. These systems were preferred because they were weather-resistant, not easily contaminated by children, and offered high storage capacities. Most rooftop rainwater harvesting systems were placed in front, sideways, and behind buildings depending on the level of disturbances and directed flow of rainwater. In terms of system management, women and children cleaned and fetched water and ensured they are well placed to tap water.

The altitudinal gradient can influence the use of rooftop rainwater harvesting systems. At the high altitudinal gradient level, the most preferred rainwater harvestings by households were plastic jerrycans and industrial plastic/metallic drums. Accessibility was central in the acquisition of systems, especially where the slopes were high and steep. So, given the challenges of accessibility the type of systems implemented by the households varied along the gradient of Mt. Elgon. Households used a variety of tools to harvest rainwater ranging from utensils to any movable kits that could store water. At moderate altitudinal gradient, the industrial plastic/metallic drums and plastic tanks were the most placed tools at this level for rainwater harvesting to supplement water supplies by the households. These systems were placed at this level to ensure the sustainability of rural rooftop rainwater harvesting but also emergency response. The systems can reduce overflows of rain events and facilitate percolation to enhance land productivity although this may not be the case in Elgon.

The trend of deploying all-year-round rooftop rainwater harvesting systems was slow between 1970 and 1990s. The uptake increased between 2000 and 2011 and rapidly picked up with sharp peaks from 2013 to 2019. The use by the households was related to changes in climate change characterized by water scarcities and shifts in seasonal calendars [19]. Households depended mostly on boreholes and open water sources such as rivers, protected springs, and shallow wells, among others. The impacts of climate change can be minimized by intensifying rooftop rainwater harvesting with good practices and designs but also regulated establishment of buildings in the study area. The systems were installed to easy domestic water availability concerns to reduce household potable water demand but also save time and labour diverted to fetch water from distant sources [32].

The dwelling roof design was majorly hip-roofed for most of the interviewed households. This design was influenced by elements of weather such as sun, wind, and humidity. It also offers a higher potential for domestic rooftop rainwater harvesting amount. Rainwater is directed towards conveyance systems from the front, sideways, and behind of the building. The household average water demand was 84 litres per day. This demand was influenced by factors such as household size and age of the household [33]. This is one of the reasons that explains why in the dry season on average the tanks were unable to meet household water demands for at least 74 days and 25 days in the wet season. The average household tank storage capacity was 307 litres. This implies that the study area has a huge potential of rainwater supply that is untapped if the storage tanks are increased in their holding capacity. This is contrary to the findings of Zhou et al. who argued that an increase in tank storage capacities may not necessarily increase water storage efficiency.

### 4.3. Amount of Rainwater Harvested by Households on the Slopes of Mt. Elgon

The potential for water saving ranges from 4.1% to 6.76% of the annual water demand with an average of 1,469 L/y per capita water saving which indicates that a person can save 4.02 litres of water daily. The high potential of households to save water is in the months between March–April and September–October periods. Rahman et al. [5] also coincided with this notion that water saving from rainwater tanks strongly correlated with the average annual rainfall received. The usefulness of harvested rainwater is enhanced when water saving and prevention of seeping techniques are employed. However, suggestions about the proposed appropriate rainwater harvesting tank sizes were beyond the scope of this study.

### 4.4. Factors Hindering the Use of Rural Rooftop Rainwater Harvesting Systems

Despite the domestic virtuous benefits of rooftop rainwater harvesting, the use and sustainability of adopted systems faced umpteen constraints. The limiting factors confronted by the households involved limited labour, high operational costs and inadequate funds, price fluctuations, unreliable rainfall, limited accessibility, scarcity of materials, land tenure, and substandard systems available on the market. Solving some of these hindrances require direct intervention by the central government. The proposed measures can include the introduction of tax waivers on rooftop rainwater harvesting systems but also closely monitoring manufactured toolkits on the market to ensure compliance with set standards. The constraints require installation of high capacity storage rooftop rainwater harvesting systems although it is also prudent to train more local artisanal manufacturers to set up the technologies. This will reduce costs and time incurred to obtain the systems in rural areas.

Much as this study was conducted at a local scale, it is of global importance to rural communities challenged with water scarcities. The Sustainable Development Goal (13 and 6) agitates for clean water and sanitation, so this study has shown the importance of rooftop rainwater harvesting in improving water supplies in a mountainous landscape. Therefore, rainwater harvesting should be one of the pillars promoted and given priority in the development and implementation of the nation and international water and agricultural related strategies.

## 5. Conclusions

This study has experienced a monotonic rainfall upward trend in March, April, September, August, and October. All the months recorded a CV of more than 30% indicating a high variability of rainfall over the slopes of Mt. Elgon. Between the 1970 and 2018 studied period, the uptake of RRWHS by the households steadily rose from 2000 up to 2018. The deployed fixed systems installed were reinforced concrete tanks, corrugated iron tanks, and plastic tanks, while the mobile systems were saucepans, metallic drums/plastic drums, jerrycans, and clay pots. The fixed rainwater harvesting systems were the most preferred at the high altitudinal gradient range, whereas the mobile systems were more opted for at the moderate and lower altitudinal gradient level. With the variability of rainfall experienced, the mean annual volume of rainwater harvested was 163,063 m^3^/yr. Rainfall data was characterized by more of the upward trend than the downward trend. The potential of households to save water ranged from 4% to 7% of the annual water demand with an average of 1,469 L/y per capita. However, the factors that affected the use of installed RRWHS were high operational costs, price fluctuations, unreliable rainfall pattern, inadequate funds, limited accessibility, and technical support.

Despite the variability of rainfall experienced, the amount of rainwater harvested is low and cannot be preserved to meet household water demands in the dry seasons. This is because the utilised systems have insufficient storage capacities, competing water uses, and poor management of the tools. However, the uptake of systems has made the households save energy, time, and costs in accessing distant water sources such as boreholes, rivers, and protected springs. With the usage of systems, households are less susceptible to waterborne diseases where, if unprotected, water sources are accessed. Therefore, the prevailing status of rainwater harvesting on the slopes of Mt. Elgon calls for urgent intervention by carrying out mass sensitisation campaigns about the values of implementing these systems. Secondly, to ignite more system uptake and use by the households, governments should introduce subsidies on the purchase of systems and increase timely access to weather forecast information.

## Figures and Tables

**Table 1:** Rainfall data (mm) and its trend.

Parameters	Jan	Feb	Mar	Apr	May	Jun	Jul	Aug	Sep	Oct	Nov	Dec
Mean	40.1	49.2	149.4	292.1	449.3	345.4	321.9	437.8	274.2	337.5	212.6	77.8
Median	35.8	36.8	110.8	216.8	433.2	308.7	263.7	353.5	211.7	274.8	172	41.8
Standard deviation	24.9	59.1	127.8	210.4	185.9	175.9	178.1	295.8	180.4	212.3	159.2	98.1
Kurtosis	2.5	10.9	7.2	3	0	−0.1	0.6	2.6	−0.4	1.7	0.2	6.3
Skewness	1.2	3.1	2.5	1.9	0.7	0.8	1	1.7	0.8	1.5	1.1	2.3
Minimum	5.1	0	22.4	104.2	192.2	81.8	51.1	131.2	35.3	101.3	48.1	0
Maximum	123.8	302.6	620.3	920	878.3	724.9	803	1341	668.9	928.4	624.3	462.4
CV (%)	62.1	120.1	85.5	72.0	41.4	50.9	55.3	67.6	65.8	62.9	74.9	126.1
Sum	1362.6	1674.4	5078.7	9931	15274.7	11744.8	10943.7	14886.3	9322.7	11476.5	7229.3	2645.1

**Table 2:** Kendall and Sen's slope test.

Months	Kendall's tau	*p* value	Sen's slope
Jan	0.12	0.33	0.37
Feb	−0.05	0.70	−0.20
Mar	0.26	**0.03**	3.11
Apr	0.45	**0.00**	7.60
May	0.04	0.75	1.33
Jun	0.01	0.98	0.08
Jul	0.15	0.23	3.31
Aug	0.23	0.06	8.01
Sep	0.27	**0.02**	7.44
Oct	0.42	**0.00**	8.87
Nov	0.14	0.24	3.02
Dec	0.07	0.57	0.53

**Table 3:** Typology of rural rooftop rainwater harvesting systems.

Rooftop rainwater harvesting systems	Description
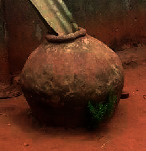	Clay pot: traditionally used to store drinking water and also used to harvest rainwater
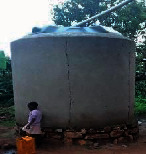	Reinforced concrete tanks: it is a conventional reinforced cement concrete technology
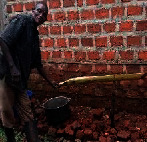	Saucepan: iron- or aluminium-made saucepans utilised to harvest rainwater
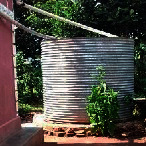	Corrugated iron tank: informally manufactured galvanised corrugated iron sheets
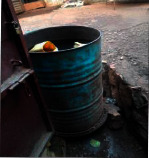	Industrial metallic/plastic drums: industrial iron drum utilised for rainwater harvesting
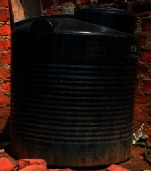	Plastic tank: factory-made plastic tanks
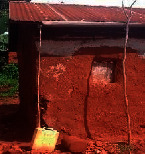	Plastic jerrycan: factory-made plastic jerrycan (normally 20 litres)

**Table 4:** Influence of altitudinal gradient ranges on the installation of rural rooftop rainwater harvesting systems (*N* = 444).

Districts	Plastic tanks	Corrugated iron tanks	Clay pots	Plastic jerrycans	Reinforced concrete tanks (%)	Industrial metallic/plastic drums	Saucepans	Altitude (m)	Slope levels
	(%)	(%)	(%)	(%)	(%)	(%)		Max	
Bududa	12	1	0	38	8	31	10	1744	High
Manafwa	17	1	2	22	5	47	6	1421	Mid
Mbale	14	1	3	43	1	30	8	1392	Low
Namisidwa	16	1	10	23	4	46	0	1367	Very low

**Table 5:** Descriptive statistics of the roof catchment area, water demand, and tank capacities.

Statistics	Maximum	Sum	Mean	Std. deviation	Variance
Roof area (sqm)	8796	88190	206.05	23.04	476.62
Daily water demand (litres)	1000	37280	84.73	3.18	66.67
*Number of days when tanks do not meet water demand*					
Dry season	160	22131	74.02	1.86	32.19
Wet season	201	6327	25.62	1.98	31.04
*Number of days when tanks meet water demand*					
Dry season	150	5528	15.10	1.24	23.70
Wet season	300	26823	63.11	2.12	43.62
Tank size storage capacity (litres)	25000	115897	307.42	77.40	1502.79

**Table 6:** Potential for water saving on the slopes of Mt. Elgon.

District	*A* (m^2^)	*R* (mm)	VR (m^3^/yr)	PWD (m^3^/y)	APPWS (%)	*P* (sampled)	PP (2019) (L/d/capita)	VRPP (m^3^/yr)
Mbale	26873	2987.35	72251.07	2887880	2.502	111	71.279	650.910
Bududa	20072	2987.35	53965.82	3237550	1.667	111	79.910	486.179
Manafwa	19038	2987.35	51185.80	3603645	1.420	111	88.946	461.133
Namisidwa	22198	2987.35	59681.81	3812425	1.565	111	94.100	537.674

## Data Availability

Data are available upon request to the corresponding author.

## References

[1] Alcamo J., Henrichs T., Rösch T. (2017). *World Water in 2025: Global Modeling and Scenario Analysis for the World Commission on Water for the 21st Century*.

[2] Mancosu N., Snyder R. L., Kyriakakis G., Spano D. (2015). Water scarcity and future challenges for food production. *Water*.

[3] Sun S., Wang Y., Liu J. (2016). Sustainability assessment of regional water resources under the DPSIR framework. *Journal of Hydrology*.

[4] Giridhar M. V. S. S., Chandra Bose A. S., Viswanadh G. K. (2013). Identification of suitable locations for rooftop rainwater harvesting structures. *International Journal of Applied Science and Engineering Research*.

[5] Rahman A., Keane J., Imteaz M. A. (2012). Rainwater harvesting in Greater Sydney: water savings, reliability and economic benefits. *Resources, Conservation and Recycling*.

[6] Bhatta G. D., Aggarwal P. K., Shrivastava A. K., Sproule L. (2016). Is rainfall gradient a factor of livelihood diversification? Empirical evidence from around climatic hotspots in Indo-Gangetic Plains. *Environment, Development and Sustainability*.

[7] Zhang S., Zhang J., Jing X., Wang Y., Wang Y., Yue T. (2018). Water saving efficiency and reliability of rainwater harvesting systems in the context of climate change. *Journal of Cleaner Production*.

[8] Kahinda J. M., Taigbenu A. E. (2011). Rainwater harvesting in South Africa: challenges and opportunities. *Physics and Chemistry of the Earth, Parts A/B/C*.

[9] Nijhof S., Jantowski B., Meerman R., Schoemaker A. (2010). Rainwater harvesting in challenging environments: towards institutional frameworks for sustainable domestic water supply. *Waterlines*.

[10] Freitas A. C. V., Aímola L., Ambrizzi T., de Oliveira C. P. (2017). Extreme intertropical convergence Zone shifts over southern maritime continent. *Atmospheric Science Letters*.

[11] Kiggundu N., Wanyama J., Mfitumukiz D. (2018). “Rainwater harvesting knowledge and practice for agricultural production in a changing climate: a review from Uganda’s perspective”. *Agricultural Engineering International: CIGR Journal*.

[12] Tripathi A. K., Pandey U. K. (2005). Study of rainwater harvesting potential of Zura village of Kutch District of Gujarat. *Journal of Human Ecology*.

[13] Tamaddun K., Kalra A., Ahmad S. (2018). Potential of rooftop rainwater harvesting to meet outdoor water demand in arid regions. *Journal of Arid Land*.

[14] Peters E. J. (2012). Drought monitoring for rooftop rainwater-harvesting systems. *Proceedings of the Institution of Civil Engineers-Water Management*.

[15] Abdulla F. A., Al-Shareef A. W. (2009). Roof rainwater harvesting systems for household water supply in Jordan. *Desalination*.

[16] Adugna D., Jensen M., Lemma B., Gebrie G. (2018). Assessing the potential for rooftop rainwater harvesting from large public institutions. *International Journal of Environmental Research and Public Health*.

[17] Salleh S. A. A., Taher T. M. (2012). Rooftop rainwater harvesting in modern cities: a case study for Sana’a City, Yemen. *Journal of Science and Technology*.

[18] Jones M. P., Hunt W. F. (2010). Performance of rainwater harvesting systems in the southeastern United States. *Resources, Conservation and Recycling*.

[19] Bomuhangi A., Nabanoga G., Namaalwa J. J., Jacobson M. G., Abwoli B., Wich S. (2016). Local communities’ perceptions of climate variability in the Mt. Elgon region, eastern Uganda. *Cogent Environmental Science*.

[20] Dawson C. (2019). Introduction to research methods 5th edition. *A Practical Guide for Anyone Undertaking a Research Project*.

[21] Uganda Bureau of Statistics (UBOS) (2018). *Uganda National Household Survey 2016/2017*.

[22] Israel G. D. (1992). *Determining Sample Size*.

[23] Boddy C. R. (2016). Sample size for qualitative research. *Qualitative Market Research: An International Journal*.

[24] Panda A., Sahu N. (2019). Trend analysis of seasonal rainfall and temperature pattern in Kalahandi, Bolangir and Koraput districts of Odisha, India. *Atmospheric Science Letters*.

[25] Mann H. B. (1945). Non-parametric tests against trend. *Econometrica*.

[26] Kendall M. (1975). *Rank Correlation Methods*.

[27] Pandit D. V. (2016). Seasonal rainfall trend analysis. *International Journal of Engineering Research and Application*.

[28] Angrill S., Petit-Boix A., Morales-Pinzón T., Josa A., Rieradevall J., Gabarrell X. (2017). Urban rainwater runoff quantity and quality—a potential endogenous resource in cities?. *Journal of Environmental Management*.

[29] Rifai S. W., Li S., Malhi Y. (2019). Coupling of El Niño events and long-term warming leads to pervasive climate extremes in the terrestrial tropics. *Environmental Research Letters*.

[30] Ongoma V., Chen H. (2017). Temporal and spatial variability of temperature and precipitation over East Africa from 1951 to 2010. *Meteorology and Atmospheric Physics*.

[31] Mugume I., Mesquita M. D., Basalirwa C. (2016). Patterns of dekadal rainfall variation over a selected region in Lake Victoria basin, Uganda. *Atmosphere*.

[32] Forrest N., Stein Z., Wiek A. (2019). Water-independent residential properties as a transformational solution to achieve water sustainability in desert cities?. *Journal of Cleaner Production*.

[33] Keshavarzi A. R., Sharifzadeh M., Kamgar Haghighi A. A., Amin S., Keshtkar S., Bamdad A. (2006). Rural domestic water consumption behavior: a case study in Ramjerd area, Fars province, I.R. Iran. *Water Research*.

